# The gamma gap predicts 4-year all-cause mortality among nonagenarians and centenarians

**DOI:** 10.1038/s41598-018-19534-4

**Published:** 2018-01-18

**Authors:** Ming Yang, Linlin Xie, Xiu Liu, Qiukui Hao, Jiaojiao Jiang, Birong Dong

**Affiliations:** 1 The Center of Gerontology and Geriatrics, West China Hospital, Sichuan University, No. 37 Guoxue Lane, Chengdu, Sichuan China; 2 The Center of Rehabilitation, West China Hospital, Sichuan University, No. 37 Guoxue Lane, Chengdu, Sichuan China

## Abstract

Recent studies have revealed the prognostic role of the gamma gap, the total serum proteins concentration minus the albumin concentration, for predicting all-cause mortality among adults. This study aims to investigate the relationship between the gamma gap and all-cause mortality among nonagenarians and centenarians via a secondary data analysis of a prospective observational study. The analysis included 801 participants (260 men and 541 women, mean age: 93.7 ± 3.5 years), 46 of which were lost at the 4-year follow-up. The mean gamma gap was 2.7 ± 0.5 g/dl. After adjusting for relevant confounders, the gamma gap was significantly associated with 4-year all-cause mortality (hazard ratio [HR] per 1-SD = 1.22, 95% confidential interval [CI]: 1.12–1.78). Using different cut-off points, the elevated gamma gap could be defined as ≥2.9, 3.0, 3.1, or 3.2 g/dl. The relevant HRs and 95% CIs of the elevated gamma gap for predicting mortality were 1.27 (1.12–1.90), 1.29 (1.03–1.78), 1.21 (1.23–1.66), and 1.26 (1.09–1.69), respectively. In conclusion, the gamma gap is an independent prognostic factor for long-term mortality in nonagenarians and centenarians. A value greater than or equal to 3.1 g/dl may define an elevated gamma gap, but further studies are required.

## Introduction

The gamma gap, the total serum proteins concentration minus the albumin concentration, has been widely used in clinical practice for several decades^1^. An elevated gamma gap is usually considered to be an indicator of infection, systemic inflammation, or malignancy^2^; however, its implications for patient prognosis are not well understood. Previous studies have indicated the potential association between an elevated gamma gap and deleterious health outcomes. For example, a previous study demonstrated that an elevated gamma gap was related to an increased risk of heart failure^3^, which was possibly the result of systemic inflammation.

A recent cohort study by Juraschek *et al*. reported that an elevated gamma gap (≥3.1 g/dl) was an independent risk factor for all-cause mortality among a large population of U.S. adults^4^. A subsequent study confirmed the effect of the gamma gap on all-cause mortality using the same study population with a longer follow-up period^5^. These findings need to be validated in different populations.

A chronic low-grade inflammation plays a key role in the process of aging^6^. Older adults, especially the oldest old, are therefore prone to chronic low-grade systemic inflammation and an elevated gamma gap^7^. Whether the gamma gap is related to an increased risk of death in nonagenarian and centenarian populations is not known. The gamma gap might serve as a valuable and cheap prognostic factor as total serum proteins and albumin (instead of other inflammatory factors, such as C-reactive protein or interleukin 6) are routinely measuredly as prat of a regular examination. Thus, we conducted this study to investigate the possible relationship between the gamma gap and all-cause mortality among a population of nonagenarians and centenarians in western China.

## Methods

### Study population

This study was a secondary data analysis of the “Project of Longevity and Aging in Dujiangyan” (PLAD), which was previous published^8,9^. In brief, all individuals (aged ≥90 years) who lived in Dujiangyan (a small city in western China) were invited to join the baseline investigation in April 2005. A total of 1115 older adults were invited, of which 870 agreed to participate. Well trained staff visited all the participants in their homes or their community centers and performed face-to-face interviews and anthropometric measurements. Biological specimens included fasting venous blood samples.

### Ethics

The Research Ethics Committee of Sichuan University (registration number: 2004081233) approved the study protocol. All the participants or their legal proxies signed the informed consents forms. All the methods in this study were in accordance with relevant guideline and regulations.

### All-cause mortality

The study population was followed up four years later, in 2009. Mortality data were collected from the local death registration database. Both positive and negative matches were then confirmed with the family members. Individuals who had no record of mortality in the database and whose family members could not be contacted were defined as “loss to follow-up” (45 individuals, 5.5%). The time to death was calculated as the time between the baseline interview and the date of death.

### Gamma gap

The measurement of total serum proteins and albumin were performed using a Synchron CX4 Pro (Beckman Coulter, Fullerton, CA). The gamma gap was calculated as “the difference between total protein and albumin (total protein – albumin)”^4^. Traditionally, an elevated gamma gap was defined as ≥4.0 g/dl; however, previous studies^2,4,10^ applied a cut-off point of ≥3.1 g/dl to define an elevated gamma gap. In this study, we defined the elevated gamma gap with multiple cut-off points between 1.7 g/dl (10^th^ percentile) and 4.4 g/dl (99^th^ percentile). We also treated the gamma gap as a continuous variable.

### Assessment of covariates

We included the following covariates: age, gender, smoking status (current smoker or not), alcohol consumption (current drinker or not), physical activity (yes/no), and body mass index (BMI, kg/m^2^).

The following chronic diseases were also included as covariates: hypertension (yes/no), cardiovascular disease (yes/no), diabetes (yes/no), chronic respiratory disease (yes/no), chronic liver disease (yes/no), stroke (yes/no), chronic kidney disease (yes/no), osteoarthritis (yes/no), and cancer (yes/no). The diagnosis of these chronic diseases was based on the formal medical records of each individual. The definitions of these diseases are listed in Supplementary Table 1.

Based on previous studies^4,5^, we also included the following covariates: total cholesterol (TC, mmol/L), triglyceride (TG, mmol/L), low-density lipoprotein cholesterol (LDL-C, mmol/L) high-density lipoprotein cholesterol (HDL-C, mmol/L), alanine aminotransferase (ALT, U/L), total bilirubin (TB, μmol/L), white blood cell count (WBC, SI), and hemoglobin (HGB, g/L). Furthermore, we included the following geriatric syndromes as covariates: self-reported falls in the previous year (yes/no), incontinence (yes/no), cognitive impairment (yes/no, assessed using Mini Mental State Examination^11^), polypharmacy (yes/no, defined as the concomitant use of five or more medications^12^), and depression (yes/no, assessed using Geriatric Depression Scale-Chinese Edition^13^).

### Statistical analyses

Baseline characteristics were presented as a proportion (%) and mean ± standard deviation (SD) of the whole study population and between elevated and normal gamma gap groups. To compare the differences between groups, the Pearson chi-squared test (or Fisher’s exact test where an expected cell count was <5) and one-way ANOVA were applied for categorical data and continuous data, respectively. Weighted Cox proportional hazard models were applied to compare the relationship between the gamma gap (as a dichotomous variable using multiple cut-off points between 1.7 and 4.4 g/dl) with all-cause mortality. Model 1 was adjusted for age, gender, smoking status, alcohol consumption, and BMI. Model 2 was further adjusted for TC, TG, HDL-C, LDL-C, ALT, TB, WBC, and HGB. We also treated the gamma gap as a continuous variable (per 1 g/dl, or per 1-SD) and reanalyzed the association between the gamma gap and all-cause mortality using the same Cox proportional hazard models listed above. Survival curves were estimated using the Kaplan–Meier method and compared using the log-rank test. The Kaplan-Meier curves and the log-rank tests were performed using MedCalc Statistical Software version 15.2 (MedCalc Software bvba, Ostend, Belgium). All other statistical analyses were performed using SPSS 20.0 (IBM, SPSS Statistics, Armonk, NY, USA). P <0.05 was considered statistically significant.

## Results

### Baseline characteristics of the study population

Participants with missing data on the gamma gap (69 individuals) were excluded in the subsequent analyses. As a result, 801 participants (260 men and 541 women) were included in the baseline analyses. All the participants were Han Chinese. The mean age of the study population was 93.7 ± 3.5 years. Among the whole study population, 180 participants (22.5%) had an elevated gamma gap (defined as ≥3.1 g/dl).

Table 1 shows the characteristics of the whole population the gamma gap values. Compared with those with a normal gamma gap, the participants with an elevated gamma gap (≥3.1 g/dl) were more likely to have higher levels of TB and WBC, but a lower level of HDL-C. The participants with an elevated gamma gap were less likely to exercise. Additionally, the participants with an elevated gamma gap appeared to be older than those with a normal gamma gap, but the difference was not statistically significant.

**Table 1:** Baseline characteristics of the whole population and corresponding gamma gap values.

	Total n (%)	Gamma gap			
		Normal (<3.1 g/dl) n (%)	Elevated (≥3.1 g/dl) n (%)	χ^2^ or F value	p
n	801	621 (77.5)	180 (22.5)	—	—
Age, year^†^	93.7 ± 3.5	93.6 ± 3.3	94.1 ± 4.0	3.680	0.055
Women	541 (67.5)	417 (67.1)	124 (68.9)	0.193	0.661
Current smokers	350 (43.6)	279 (44.9)	71 (39.4)	1.705	0.192
Current alcohol drinkers	211 (26.3)	159 (25.6)	52 (28.9)	0.776	0.378
Exercise habits^*^	300 (38.2)	244 (40.2)	56 (31.5)	4.126	0.035
BMI, kg/m^2 †^	19.3 ± 3.5	19.2 ± 3.4	19.4 ± 3.6	0.149	0.700
Chronic disease					
Hypertension	330 (41.2)	253 (40.7)	77 (42.8)	0.239	0.625
Cardiovascular disease	72 (9.0)	50 (8.1)	22 (12.2)	2.967	0.085
Diabetes	18 (2.2)	13 (2.1)	5 (2.8)	0.298	0.585
Chronic respiratory disease	176 (22.0)	130 (20.9)	46 (25.6)	1.739	0.187
Chronic liver disease	53 (6.6)	37 (6.0)	16 (8.9)	1.940	0.164
Stroke	48 (6.0)	35 (5.6)	13 (7.2)	0.623	0.430
Chronic kidney disease	36 (4.6)	26 (4.2)	10 (5.6)	0.609	0.435
Osteoarthritis	312 (39.0)	250 (40.3)	62 (34.4)	1.983	0.159
Cancer	13 (1.6)	8 (1.3)	4 (2.2)	—	0.483
Geriatric syndromes					
Falls in the previous 12 months	429 (53.5)	341 (54.9)	88 (48.9)	2.035	0.154
Incontinence	83 (10.4)	61 (9.8)	22 (12.2)	0.865	0.352
Cognitive impairment^*^	187 (24.0)	150 (24.6)	36 (20.6)	1.178	0.270
Polypharmacy	162 (20.2)	118 (19.0)	44 (24.4)	2.562	0.109
Depression	180 (22.5)	131 (21.1)	49 (27.2)	3.007	0.083
Gamma gap, g/dl^†^	2.7 ± 0.5	2.5 ± 0.3	3.4 ± 0.4	891.168	<0.001
TC, mmol/L^†^	4.5 ± 1.7	4.7 ± 2.1	4.0 ± 0.8	0.483	0.487
TG, mmol/L^†^	1.3 ± 0.7	1.2 ± 0.7	1.3 ± 0.8	0.768	0.381
LDL-C, mmol/L^†^	2.3 ± 0.9	2.3 ± 1.0	2.2 ± 0.6	1.439	0.231
HDL-C, mmol/L^†^	1.6 ± 0.7	1.6 ± 0.8	1.5 ± 0.3	4.085	0.034
ALT, U/L^†^	11.4 ± 9.7	11.4 ± 9.5	11.7 ± 10.2	0.197	0.658
TB, μmol/L^†^	17.2 ± 5.0	18.2 ± 6.4	13.8 ± 11.3	0.138	0.711
WBC, SI^†^	5.8 ± 1.6	5.7 ± 1.6	7.1 ± 1.8	9.131	0.003
HGB, g/L^†^	113.2 ± 15.5	113.3 ± 15.3	112.9 ± 16.3	0.128	0.721

^†^Data are presented as the mean ± standard deviation (SD). *n = 785, because of missing data on exercise habits and/or the MMSE.

One-way ANOVA was used for the continuous variables, and the Pearson chi-squared test or Fisher’s exact test (where an expected cell count was <5) was used for the categorical variables. During testing, p < 0.05 indicates statistical significance.

ALT, alanine aminotransferase; BMI, body mass index; HDL-C, high-density lipoprotein cholesterol; HGB, hemoglobin; LDL-C, low-density lipoprotein cholesterol; MMSE, Mini-Mental State Examination; TB, total bilirubin; TC, total cholesterol; TG, triglyceride; WBC, white blood cell count.

### Association between the gamma gap and 4-year all-cause mortality

In the included population, 36 participants were lost during the 4-year follow-up, and therefore, 765 participants (249 men and 516 women) were included in the survival analyses. During the 4-year follow-up, 139 men (55.8%) and 269 women (52.1%) died (p = 0.338). The mortality of the participants with an elevated gamma gap (defined as ≥3.1 g/dl) was significantly higher than those with a normal gamma gap (61.9% vs. 50.8%, p = 0.009).

When the gamma gap was treated as a continuous variable, a higher gamma gap was independently associated with a higher risk of 4-year all-cause mortality after adjusting for the relevant confounders (HR per 1 g/dl = 1.09, 95% CI: 1.01–1.69; HR per 1-SD = 1.22, 95% CI: 1.12–1.78) (Table 2).

**Table 2:** Association between the gamma gap and all-cause mortality according to Cox Regression Models adjusted for potential confounders.

	Model 1, HR (95% CI)	Model 2, HR (95% CI)
Gamma gap, continuous variable (per 1 g/dl)	**1.19 (1.02, 1.67)**	**1.09 (1.01, 1.69)**
Gamma gap, continuous variable (per 1-SD)	**1.32 (1.06, 1.76)**	**1.22 (1.12, 1.78)**
Gamma gap, dichotomized with different cut-off points		
≥1.7 g/dl	0.96 (0.89, 1.45)	0.94 (0.86, 1.55)
≥1.8 g/dl	0.98 (0.95,1.02)	0.96 (0.92, 1.04)
≥1.9 g/dl	1.05 (0.96, 1.17)	0.99 (0.94, 1.12)
≥2.0 g/dl	1.12 (0.98, 1.23)	1.09 (0.91, 1.19)
≥2.1 g/dl	1.18 (0.87, 1.62)	1.10 (0.90, 1.67)
≥2.2 g/dl	1.14 (0.83, 1.87)	1.09 (0.89, 1.71)
≥2.3 g/dl	1.09 (0.78, 1.91)	1.07 (0.80, 1.88)
≥2.4 g/dl	1.19 (0.92, 1.99)	1.09 (0.94, 1.91)
≥2.5 g/dl	1.23 (0.95, 1.86)	1.17 (0.93, 1.77)
≥2.6 g/dl	1.25 (0.98, 1.71)	1.19 (0.95, 1.69)
≥2.7 g/dl (the median value)	**1.33 (1.09, 1.88)**	1.29 (0.97, 1.79)
≥2.8 g/dl	**1.35 (1.04, 1.73)**	1.31 (1.00, 1.70)
≥2.9 g/dl	**1.32 (1.10, 1.99)**	**1.27 (1.12, 1.90)**
≥3.0 g/dl	**1.33 (1.05, 1.92)**	**1.29 (1.03, 1.78)**
≥3.1 g/dl (the previously reported cut-off point)	**1.25 (1.08, 1.57)**	**1.21 (1.13, 1.66)**
≥3.2 g/dl	**1.34 (1.11, 1.63)**	**1.26 (1.09, 1.69)**
≥3.3 g/dl	**1.37 (1.09, 1.78)**	1.34 (0.97, 1.86)
≥3.4 g/dl	**1.61 (1.20, 1.98)**	1.53 (0.99, 1.87)
≥3.5 g/dl (the 95^th^ percentile)	**1.79 (1.13, 2.43)**	1.68 (0.94, 2.12)
≥3.6 g/dl	**1.76 (1.09,2.32)**	1.54 (0.97, 2.03)
≥3.7 g/dl	**1.81 (1.17, 3.01)**	1.69 (0.91, 1.98)
≥3.8 g/dl	**1.78 (1.03, 3.65)**	1.61 (0.92, 3.47)
≥3.9 g/dl	**1.85 (1.01, 3.32)**	1.63 (0.95, 3.56)
≥4.0 g/dl (the traditional definition)	2.01 (0.99, 3.87)	1.98 (0.91, 3.71)
≥4.1 g/dl	2.09 (0.96, 3.14)	2.00 (0.89, 3.03)
≥4.4 g/dl (the 99^th^ percentile)	2.12 (0.87, 3.91)	1.96 (0.86, 3.82)

Bold represents statistical significance.

Model 1: adjusted for age, gender, smoking status, alcohol drinking, and BMI. Model 2: adjusted for age, gender, smoking status, alcohol drinking, BMI, TC, TG, HDL-C, LDL-C, ALT, TB, WBC, and HGB.

ALT, alanine aminotransferase; BMI, body mass index; CI, confidential interval; HDL-C, high-density lipoprotein cholesterol; HGB, hemoglobin; HR, hazard ratio; LDL-C, low-density lipoprotein cholesterol; TB, total bilirubin; TC, total cholesterol; TG, triglyceride; WBC, white blood cell count.

We evaluated different cut-off points to define an elevated gamma gap. After adjusting for the relevant confounders, an elevated gamma gap was significantly associated with 4-year all-cause mortality when defined as ≥2.9 g/dl (HR = 1.27, 95% CI: 1.12–1.90), ≥3.0 g/dl (HR = 1.29, 95% CI: 1.03–1.78), ≥3.1 g/dl (HR = 1.21, 95% CI: 1.13–1.66), or ≥3.2 g/dl (HR = 1.26, 95% CI 1.09–1.69). The traditional value used to define an elevated gamma gap ( ≥4.0 g/dl) was not significantly associated with 4-year all-cause mortality after full adjustment (HR = 1.98, 95% CI: 0.91–3.71) (Table 2).

**Figure 1:**
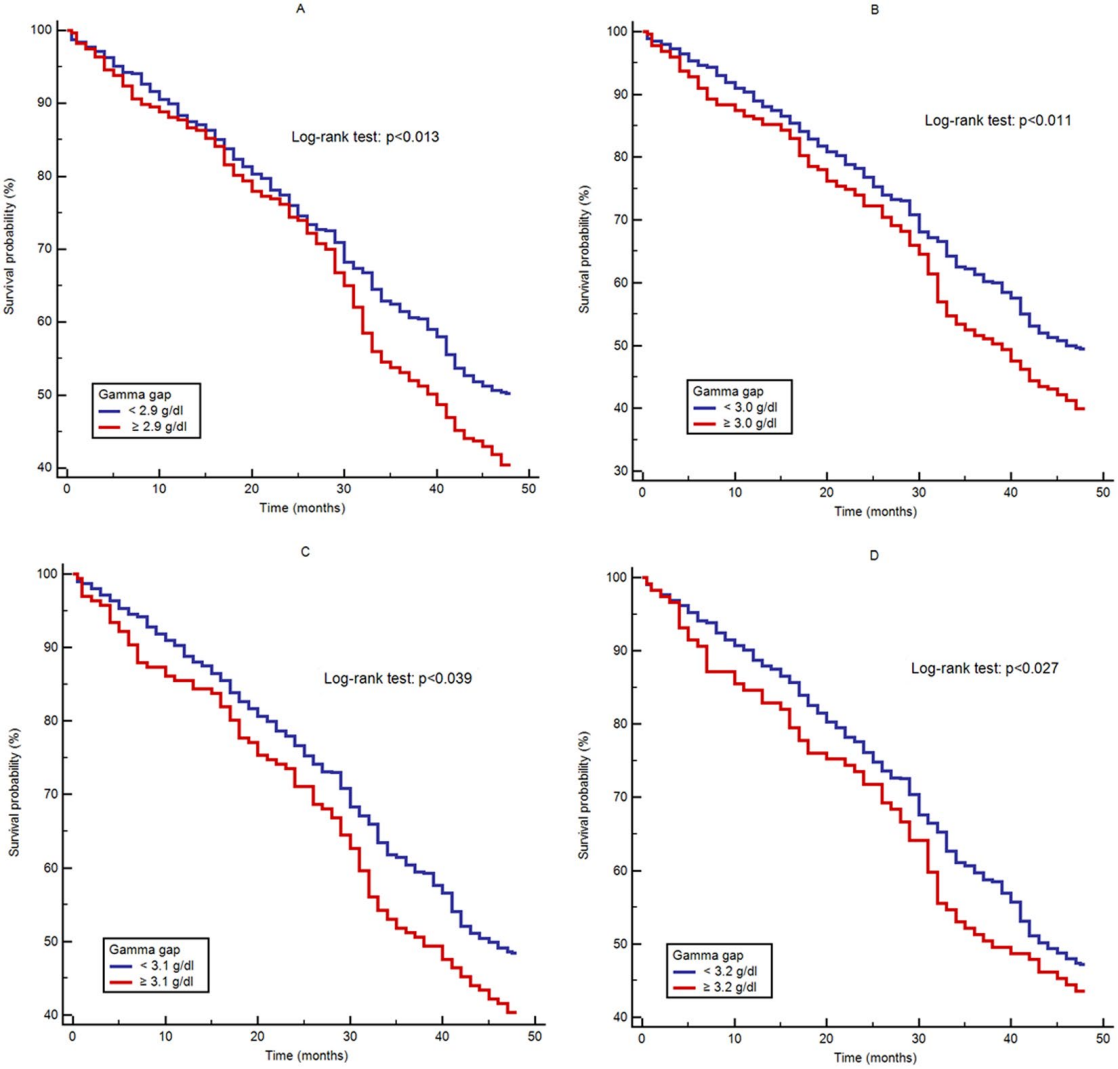
Survival curves of the participants with or without an elevated gamma gap using different cut-off points: (**A**) ≥2.9 g/dl, (**B**) ≥3.0 g/dl, (**C**) ≥3.1 g/dl, and (**D**) ≥3.2 g/dl.

Figure 1 shows the Kaplan-Meier survival curves of the participants with or without an elevated gamma gap defined by different cut-off points. The log-rank test indicated that the survival curve of the participants with an elevated gamma gap was significantly different compared to those with a normal gamma gap, irrespective of which cut-off point was applied.

## Discussion

The gamma gap is a useful marker of inflammation, malignancies, and other diseases^2^; however, its role as a prognostic factor for mortality is not clear. Our study demonstrated that the gamma gap is an independent predictor of all-cause mortality in a study population of nonagenarians and centenarians after a 4-year follow-up. This finding is in accordance with two previous studies with a large sample size^4,5^. These studies used the data from the National Health Nutrition Examination Survey (NHANES), and the mean ages of the study populations were 41.8 and 46.0 years. Our study expanded the potential prognostic role of the gamma gap for predicting all-cause mortality from nonagenarians and centenarians.

Aging itself is closely associated with chronic low-grade inflammation^6,14^. This chronically activated inflammation with advancing age has been termed as “inflamm-aging”^15^. Inflamm-aging has been identified as a risk factor for many age-related diseases (e.g., Alzheimer’s disease, cardiovascular disease, frailty, and sarcopenia) and mortality among older adults^6^. Therefore, in theory, the gamma gap (as a nonspecific inflammatory marker) should be increased in our study population. In contrast, using the same cut-off point, participants with an elevated gamma gap among our study population (22.5%) was lower than younger adults in a previous study (31.7%)^5^. One possible reason for this finding might be selective survival bias.

Loprinzi *et al*.^5^ recently reported that physical activity might play a beneficial role for reducing all-cause mortality among people with an elevated gamma gap. In addition, many previous studies have revealed the relationship between physical activity and reducing the risk of mortality in different populations^16,17^. For example, in a prospective cohort study with 204,542 adults aged 45–75 years, an inverse dose-response relationship was identified between physical activity and mortality^18^. We did not measure physical activity among our study participants; however, our study demonstrated that the participants with an elevated gamma gap were less likely to exercise. One of the proposed mechanisms responsible for this finding is that exercise can reduce systemic inflammation^14,19^.

One strength of our study is the exploration of optimal cut-off points to define the elevated gamma gap in nonagenarians and centenarians. We found that by using the cut-off points of 2.9, 3.0, 3.1 or 3.2 g/dl (instead of the traditional value of 4.0 g/dl), the elevated gamma gap had similar HRs for predicting long-term mortality in our study population. Previous studies also demonstrated that the cut-off point of 3.1 g/dl was suitable to define an elevated gamma gap in adults^2,4,5,10,20^. Putting these findings together, we suggest that an elevated gamma gap could also be defined as ≥3.1 g/dl in the oldest adults.

Our study has some limitations. First, because the gamma gap is considered a marker of inflammation, we need to adjust for other inflammatory factors as potential confounders such as C-reactive protein (CRP) or interleukin (IL)-6. We did not include these factors in the Cox proportional hazard models due to a lack of relevant data. Second, as with all cohort studies, selective survival prior to entry in the cohort needs to be considered a potential bias.

## Conclusion

The gamma gap is an independent prognostic factor for 4-year all-cause mortality among a study population of nonagenarians and centenarians. The elevated gamma gap can be defined as ≥3.1 g/dl in the oldest adults. Further prospective studies are required to validate these findings in different ethnic populations with various diseases, especially in older adults. The relationship between the gamma gap and disease-specific mortality also needs to be explored.

## Electronic supplementary material

Supplementary Table 1
