# Supplementary material for: The gamma gap predicts 4-year all-cause mortality among nonagenarians and centenarians
Source: Sci Rep. 2018 Jan 18;8:1046. doi: 10.1038/s41598-018-19534-4 (PMC5773485; doi:10.1038/s41598-018-19534-4)
Supplement: Supplementary file 1 — Supplementary Table 1 [file 41598_2018_19534_MOESM1_ESM.doc]

# Title page

**Title:**

The gamma gap predicts 4-year all-cause mortality among nonagenarians and centenarians

**Authors names and affiliations**

Ming Yang ^1^, MD; Linlin Xie ^1^, MSN; Xiu Liu ^1^, MSN; Qiukui Hao ^1^, MD; Jiaojiao Jiang ^2^, MSS; Birong Dong ^1^, MD

1. The Center of Gerontology and Geriatrics, West China Hospital, Sichuan University, No. 37 Guoxue Lane, Chengdu, Sichuan, China.

2. The Center of Rehabilitation, West China Hospital, Sichuan University, No. 37 Guoxue Lane, Chengdu, Sichuan, China.

**Corresponding author and contact details:**

Associated prof. Ming Yang will handle correspondence at all stages of refereeing, publication, and post-publication. Address: The Center of Gerontology and Geriatrics, West China Hospital of Sichuan University, 37 Guoxue Lane, Chengdu, China. Phone: +86 28 8542 2326. Fax: +86 28 8542 2321. Email: [yangmier@gmail.com](mailto:yangmier@gmail.com)

**Supplementary Table 1. The definitions of the chronic diseases in this study**

| **Chronic diseases** | **Definitions** |
| --- | --- |
| Hypertension | The resting blood pressure is persistently at or above 140/90 mmHg. OR the subject has a medical history of hypertension and is currently received anti-hypertensive therapy. |
| Cardiovascular disease | A cluster of diseases including coronary artery diseases, heart failure, hypertensive heart disease, cardiomyopathy, rheumatic heart disease, arrhythmia, valvular heart disease, and peripheral artery disease. |
| Diabetes | One of the following criteria:   - Fasting plasma glucose level ≥ 7.0 mmol/L - Plasma glucose ≥ 11.1 mmol/L two hours after a 75 g oral glucose load - Symptoms of high blood sugar (e.g., polyuria, polydipsia, and polyphagia) and casual plasma glucose≥ 11.1 mmol/L - Glycated hemoglobin ≥ 6.5% - Currently received antidiabetic therapy |
| Chronic respiratory disease | A cluster of diseases including chronic obstructive pulmonary disease, asthma, pulmonary arterial hypertension, and interstitial lung disease. |
| Chronic liver disease | A cluster of diseases including chronic hepatitis, alcoholic liver disease, cirrhosis, and non-alcoholic fatty liver disease, |
| Stroke | Both ischemic stroke and hemorrhagic stroke are included. However, transient ischemic attack (TIA) is excluded. |
| Chronic kidney disease | A glomerular filtration rate (GFR) <60 ml/min/1.73 m2 for more than 3 months. |
| Osteoarthritis | The diagnosis is based on symptoms (e.g., joint pain and stiffness), and X-ray. Most of our participants suffered from knee osteoarthritis. |
| Cancer | Cancer of any type, such as lung cancer, liver cancer, lymphoma, leukemia, prostate cancer, colon cancer, breast cancer, etc. Benign tumors are excluded. |
